# Verification of Laser Heterodyne Interferometric Bench for Chinese Spaceborne Gravitational Wave Detection Missions

**DOI:** 10.34133/research.0302

**Published:** 2024-02-14

**Authors:** Xin Xu, Heshan Liu, Yidong Tan

**Affiliations:** ^1^ Department of Precision Instrument, Tsinghua University, Beijing 100084, China.; ^2^ State Key Laboratory of Precision Measurement Technology and Instrument, Tsinghua University, Beijing 100084, China.; ^3^ National Microgravity Laboratory, Institute of Mechanics, Chinese Academy of Sciences, Beijing 100190, China.

## Abstract

Construction of laser heterodyne interferometric bench to measure tiny translation and tilt with picometer- and nanoradian-level sensitivity in the millihertz band is critical for the success of spaceborne gravitational wave detection, including the LISA, Taiji, and Tianqin missions. In this paper, we report on the construction and testing of a laser heterodyne interferometric bench that contains two optical path designs, the dual-beam heterodyne interferometry and the polarization-multiplexing heterodyne interferometry. The measurement sensitivity of translation and tilt reaches below 3 pm/Hz^1/2^ and 12 nrad/Hz^1/2^ for frequencies above 10 mHz, respectively. As a technical verification platform, stabilization loops of amplitude and phase and coherence analysis are also conducted through the bench. Furthermore, we demonstrate initial implements of phase-locking technology and multiple degree of freedom measurements as the extended applications of the constructed bench. The achieved results show that the laser interferometric bench would serve as an excellent experimental platform for the technology demonstration and verification of future Chinese spaceborne gravitational wave detection.

## Introduction

Spaceborne gravitational wave detection has recently emerged as a research hotspot, offering great potential to enhance our understanding of the universe, including the laser interferometer space antenna (LISA), Taiji, and Tianqin missions [1–4]. The leading approach to these missions involves the construction of long-arm laser interferometers to measure the minuscule distortions between two freely falling test masses caused by passing gravitational waves [5]. While ground-based laser interferometers face limitations due to seismic noise and arm length, spaceborne arrangements overcome these challenges and open up a low-frequency gravitational wave detection window. In general, the current optical metrology setup features three main functional interferometers: the science interferometer (for monitoring the distance change between the local and remote optical benches), the test mass interferometer (for monitoring the distance change between the test mass and the optical bench), and the reference interferometer (for monitoring the front-end common-mode noise) [6]. The primary requirement of spaceborne optical metrology is to achieve a measurement sensitivity of ~1 pm/Hz^1/2^ of translation and ~1 nrad/Hz^1/2^ of tilt using laser heterodyne interferometry and differential wavefront sensing.

Design concepts for interferometers aimed at detecting gravitational waves in space were initially determined in the late 20th and early 21st centuries [7,8]. In subsequent years, an integrated optical bench containing four functional interferometers was designed, constructed, and tested in the LISA Pathfinder mission [9–12]. The latest experimental results from LISA Pathfinder in space demonstrate a measurement sensitivity of 0.032 pm/Hz^1/2^ at 1 Hz, representing the best performance of the optical interferometric bench so far for the spaceborne gravitational wave detection [13,14]. Besides the translation and tilt measurements [15–17], precious researches on interferometric benches for other technology verifications have been constructed and tested, such as tilt-to-length coupling [18,19], spaceborne telescope testing [20], digital phase-locking loop [21], coefficient measurement of thermal expansion [22], and multiple degree of freedom sensing [23]. China’s research on heterodyne interferometers for spaceborne gravitational wave detection started in the 2010s, mainly from Taiji and Tianqin missions. In 2020, a laser interferometer prototype has been designed and constructed, achieving a measurement sensitivity below 6 pm/Hz^1/2^ within the frequency range of 10 mHz to 1 Hz [24]. Additionally, optical interferometric benches developed based on optical-bonding technology have been constructed and tested in space [25–28]. Yan et al. [23] constructed a laser heterodyne interferometric bench for the six-degree-of-freedom readout, which offers an alternative for the test mass motion monitor and expands the applications of the optical bench. Nevertheless, the optical interferometers of China for spaceborne gravitational wave detection, to the best of our knowledge, could not fully reach the sensitivity goal of ~1 pm/Hz^1/2^ in terms of translation measurement. Additionally, research studies on tilt readout using quadrant photodetectors compared to translation are still limited, thus requiring further investigations. Furthermore, in order to promote the interferometer stability, constructions of laser interferometric benches are currently based on one-piece optical-bonding baseplate, which is expensive and challenging to modify; thus, a flexible experimental platform is essential during the technical validation period. Overall, future spaceborne missions for gravitational wave research call for an improved optical interferometric bench with ultra-high sensitivity and flexibility to reach the required measurement sensitivity and verify relevant key technologies.

This paper aims to address the challenges of the required optical metrology in the future China-leading spaceborne gravitational wave detection missions, particularly the measurement sensitivity for translation and tilt readout in the millihertz band. Additionally, other interferometric technologies are also worth of notice and need a platform to investigate, such as noise analysis and mitigation, phase locking, multiple degree of freedom sensing, and so on. Therefore, we design and experimentally construct an ultra-sensitive interferometric bench and conduct experimental tests in a vacuum chamber to evaluate its measurement sensitivity, which are shown in the “Results” section. The ability for technical verification and exploration through the constructed bench has been demonstrated in the “Discussion and Conclusion” section, including the stabilization loops of amplitude and phase, coherence analysis, phase-locking technology, and multiple degree of freedom measurements. In a sense, the presented laser interferometric bench might be a promising candidate for the valid technology demonstration and verification of Chinese spaceborne gravitational wave detection missions, which is concluded in the “Discussion and Conclusion” section. Details of materials and methods for the experiments are introduced in the “Materials and Methods” section.

## Results

### Concept and principle

**Fig. 1.**
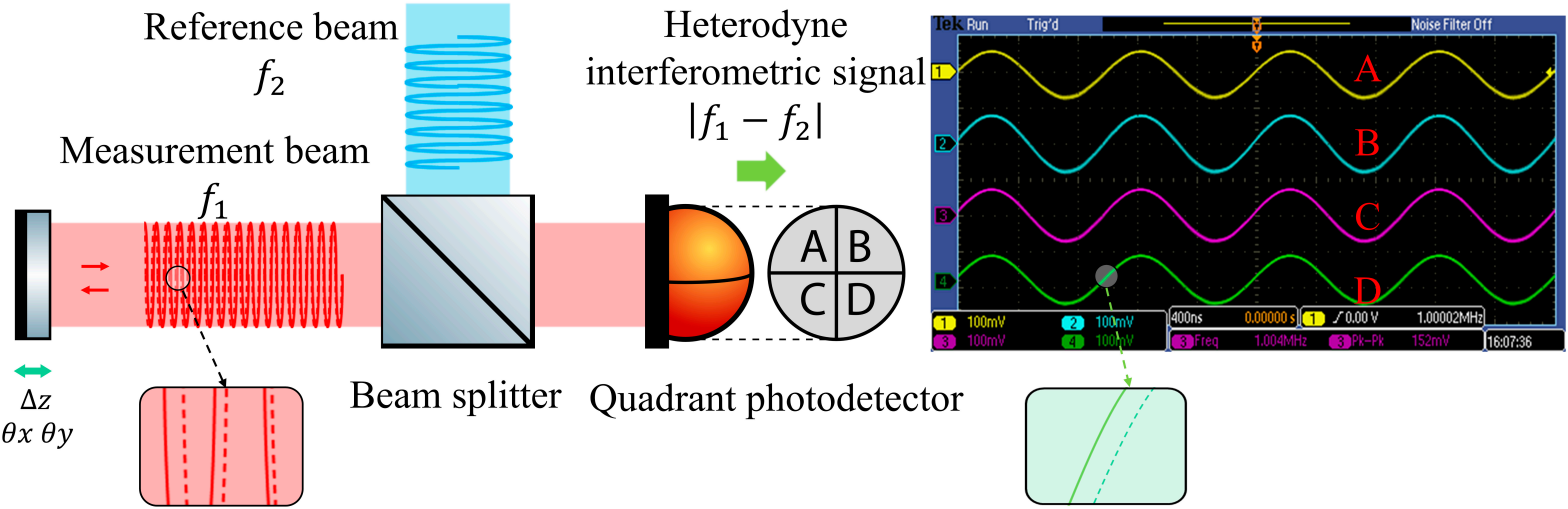
Conceptual illustration of laser heterodyne interferometry and differerential wavefront sensing.

Laser heterodyne interferometry is a technique that enables high-precision and high-sensitivity measurements of tiny distance changes between two points. As depicted in Fig. 1, two laser beams of slightly different frequencies are utilized and combined to generate heterodyne interferometric signals, from which the motion information of the target can be extracted by analyzing the phase changes [29]. When the target (the measured reflector) moves along the direction of beam propagation, the phase will vary as the dashed line shows. Specifically, a one-cycle phase variation of the heterodyne interferometric signals corresponds to a translation change of half a wavelength (optical path length with two subdivisions, for example here). While the target rotates, the wavefront of the measurement beam deviates from the reference beam, leading to phase fluctuations of the interferometric beat. Due to the spatial distance between the four-quadrant detector elements, the tilt of the target in the horizontal and vertical direction can be obtained by analyzing the phase differences of four interferometric outputs using the quadrant photodetector. This angle measurement technique is called differential wavefront sensing, and its core device is the quadrant photodetector [30].

**Fig. 2.**
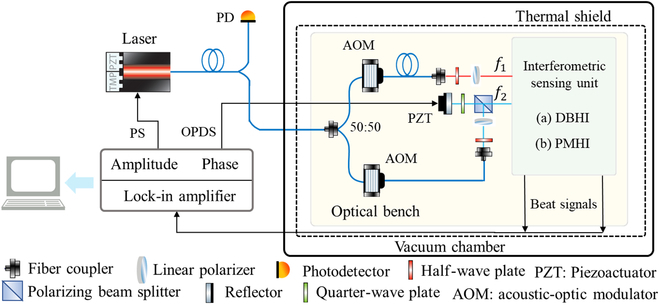
Setup of the laser heterodyne interferometric bench. PS, power stabilization; OPDS, optical path difference stabilization; DBHI, dual-beam heterodyne interferometry; PMHI, polarization-multiplexing heterodyne interferometry.

**Fig. 3.**
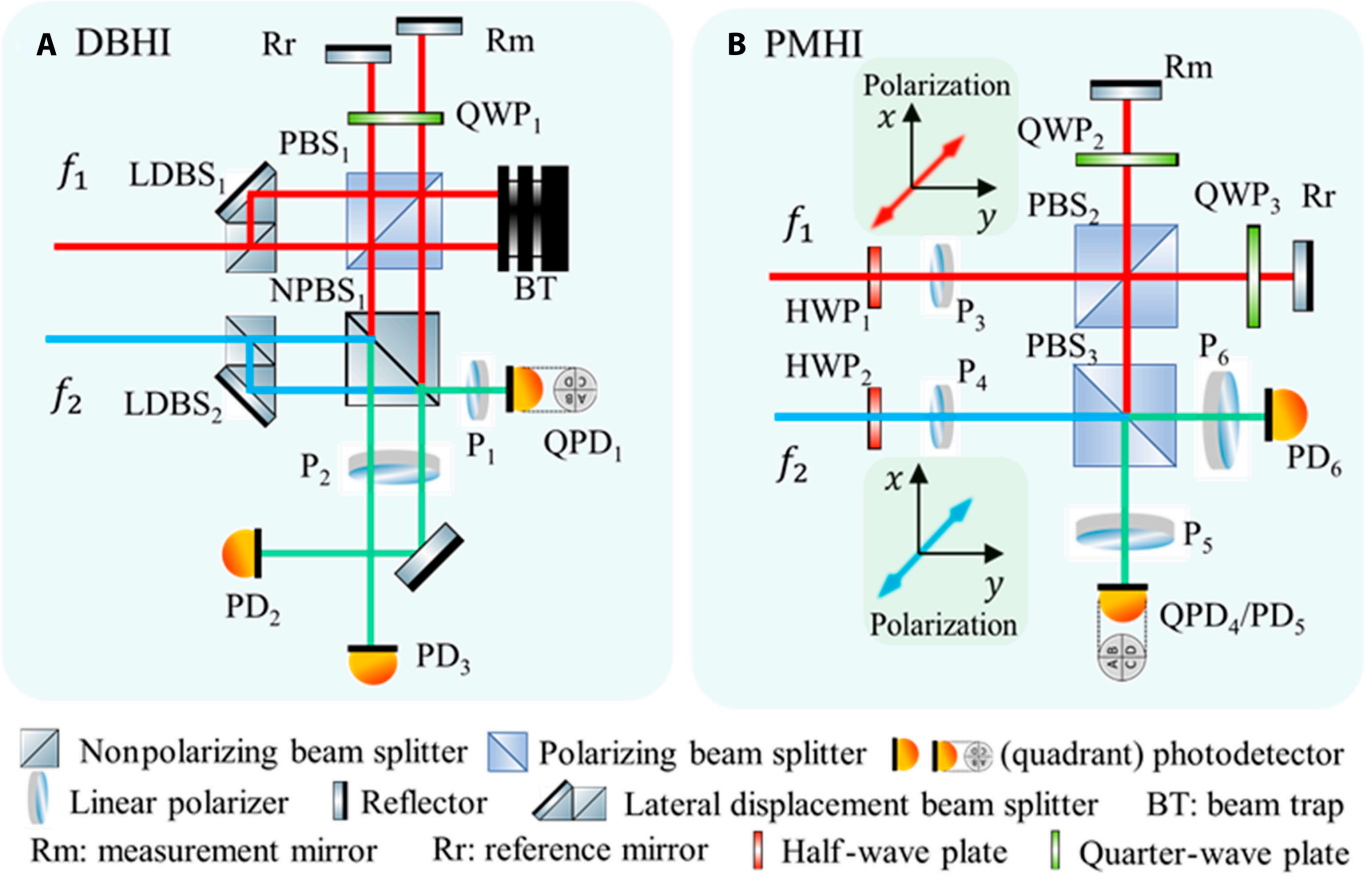
Two optical path designs of the constructed laser interferometric bench. (A) DBHI. (B) PMHI.

By combining these two techniques, translation and tilt measurement of the test mass can be both achieved in the spaceborne gravitational wave detection. To verify this, a heterodyne interferometric bench is constructed based on previous researches [15–17]. The interferometric system, illustrated in Fig. 2, consists of a laser source, an optical bench, and a signal management unit. Single-frequency laser is transmitted through a single-mode polarization-maintaining fiber into the optical bench, where it is frequency-shifted by a pair of acousto-optic modulators. The two beams, with a frequency difference of 1 MHz and almost equal optical path length and power, are injected into the spatial interferometric part as the measurement beam (*f*_1_) and the reference beam (*f*_2_). Figure 3 shows the specific layout of the interferometric sensing unit, containing two optical path designs, which are respectively named as dual-beam heterodyne interferometry (DBHI) and polarization-multiplexing heterodyne interferometry (PMHI).

For DBHI, two parallel beams, directed by lateral displacement beam splitters from the measurement beam, are incident on the reflectors (Rm/Rr) and are then combined with the reference beams using a nonpolarization beam splitter. One quadrant photodetector (QPD_1_) and two single-element photodetectors (PD_2/3_) are used to obtain the heterodyne interferometric signals, which are sent to the lock-in amplifier for the amplitude and phase acquisition [31]. Based on the laser heterodyne interferometry and differential wavefront sensing, DBHI can measure the translation and the tilt of the reflector (Rm) with a common-path design.

For PMHI, the polarization states of the incident beams are guaranteed by a half-wave plate and a linear polarizer. Then, the measurement beams are separated into two parts by the polarizing beam splitter (PBS_2_) onto the reflectors (Rm/Rr). After transmitting through the PBS_2_, the measurement beams are combined with the reference beams using a polarizing beam splitter (PBS_3_). Similarly, one quadrant photodetector (QPD_4_) and two single-element photodetectors (PD_5/6_) are used to obtain the heterodyne interferometric signals, which are sent to the lock-in amplifier for the amplitude and phase acquisition. Based on the laser heterodyne interferometry and differential wavefront sensing, PMHI can measure the translation and the tilt of the reflector (Rm) with a polarization-multiplexing design.

The specific optical paths are listed in Tables 1 and 2. The translation and tilt readout of the interferometric bench for DBHI can be mathematically expressed as:

$$\Delta L_{M-\text{DBHI}}=\frac{\lambda }{4\pi }\left(\varphi_{{\mathrm{PD}}_{2}}-\varphi_{\mathrm{AOM}-\text{drivers}}\right)$$ (1)

$$\Delta L_{R-\text{DBHI}}=\frac{\lambda }{4\pi }\left(\varphi_{{\mathrm{PD}}_{3}}-\varphi_{\mathrm{AOM}-\text{drivers}}\right)$$ (2)

$$\begin{array}{c} \Delta L_{\text{DBHI}}=\Delta L_{M-\text{DBHI}}\;-\Delta L_{R-\text{DBHI}}=\\ \frac{\lambda }{4\pi }\left(\varphi_{PD_{2}}-\varphi_{PD_{3}}\right)=\frac{\lambda }{4\pi }\left(\varphi_{QPD_{1A}}-\varphi_{PD_{3}}\right) \end{array}$$ (3)

$$\Delta \theta_{\mathrm{db}}=\frac{\Delta L_{M}-\Delta L_{R}}{D_{\mathrm{db}}}$$ (4)

$$\Delta \theta_{v-\text{DBHI}}=\frac{\lambda }{4\sqrt{2\pi }d}\left(\varphi_{{\mathrm{QPD}}_{1A}}-\varphi_{{\mathrm{QPD}}_{1B}}-\varphi_{{\mathrm{QPD}}_{1C}}+\varphi_{{\mathrm{QPD}}_{1D}}\right)$$ (5)

$$\Delta \theta_{h-\text{DBHI}}=\frac{\lambda }{4\sqrt{2\pi }d}\left(\varphi_{{\mathrm{QPD}}_{1A}}+\varphi_{{\mathrm{QPD}}_{1B}}-\varphi_{{\mathrm{QPD}}_{1C}}-\varphi_{{\mathrm{QPD}}_{1D}}\right)$$ (6)

**Table 1.** Detailed optical paths for the dual-beam heterodyne interferometry

DBHI	Measurement interferometer	Path1_m	*f*_1_→LDBS_1_→PBS_1_→QWP_1_→Rm→QWP_1_→PBS_1_→NPBS_1_→P_1_(P_2_)→QPD_1_ (PD_2_)
		Path1_r	*f*_2_→LDBS_2_→NPBS_1_→P_1_(P_2_)→QPD_1_ (PD_2_)
	Reference interferometer	Path2_m	*f*_1_→LDBS_1_→PBS_1_→QWP_1_→Rr→QWP_1_→PBS_1_→NPBS_1_→P_2_→PD_3_
		Path2_r	*f*_2_→LDBS_2_→NPBS_1_→P_2_→PD_3_

**Table 2.** Detailed optical paths for the polarization-multiplexing heterodyne interferometry

PMHI	Measurement interferometer	Path3_m	*f*_1_→HWP_1_→P_3_→PBS_2_→QWP_2_→Rm→QWP_2_→PBS_2_→PBS_3_→P_5_→QPD_4_ (PD_5_)
		Path3_r	*f*_2_→HWP_2_→P_4_→PBS_3_→P_5_→QPD_4_ (PD_5_)
	Reference interferometer	Path4_m	*f*_1_→HWP_1_→P_3_→PBS_2_→QWP_3_→Rr→QWP_3_→PBS_2_→PBS_3_→P_6_→PD_6_
		Path4_r	*f*_2_→HWP_2_→P_4_→PBS_3_→P_6_→PD_6_

Note that the reference signal here can be obtained from the RF-mixer output of AOM drivers for the optical path length readout (phase difference between the signals from PD_2/5_ and AOM drivers, see Eq. 1/7), or from the reference interferometer for the final interferometric readout (phase difference between the signals from QPD_1_/PD_2_ and PD_3_ of DBHI or from QPD_4_/PD_5_ and PD_6_ of PMHI, see Eq. 3/9). The reference interferometer has an almost identical optical path to the measurement interferometer, allowing for the effective elimination of common-mode noises, such as differential phase fluctuation originating from the optical fibers, modulators, or environmental perturbation.

The translation and tilt readout of the interferometric bench for PMHI can be mathematically expressed as:

$$\Delta L_{M-\text{PMHI}}=\frac{\lambda }{4\pi }\left(\varphi_{{\mathrm{PD}}_{5}}-\varphi_{\mathrm{AOM}-\text{drivers}}\right)$$ (7)

$$\Delta L_{R-\text{PMHI}}=\frac{\lambda }{4\pi }\left(\varphi_{{\mathrm{PD}}_{6}}-\varphi_{\mathrm{AOM}-\text{drivers}}\right)$$ (8)

$$\begin{array}{c} \Delta L_{\text{PMHI}}=\Delta L_{M-\text{PMHI}}-\Delta L_{R-\text{PMHI}}=\\ \frac{\lambda }{4\pi }\left(\varphi_{{\mathrm{PD}}_{5}}-\varphi_{{\mathrm{PD}}_{6}}\right)=\frac{\lambda }{4\pi }\left(\varphi_{{\mathrm{QPD}}_{4A}}-\varphi_{{\mathrm{PD}}_{6}}\right) \end{array}$$ (9)

$$\Delta \theta_{v-\text{PMHI}}=\frac{\lambda }{4\sqrt{2\pi }d}\left(\varphi_{{\mathrm{QPD}}_{4A}}-\varphi_{{\mathrm{QPD}}_{4B}}-\varphi_{{\mathrm{QPD}}_{4C}}+\varphi_{{\mathrm{QPD}}_{4D}}\right)$$ (10)

$$\Delta \theta_{h-\text{PMHI}}=\frac{\lambda }{4\sqrt{2\pi }d}\left(\varphi_{{\mathrm{QPD}}_{4A}}+\varphi_{{\mathrm{QPD}}_{4B}}-\varphi_{{\mathrm{QPD}}_{4C}}-\varphi_{{\mathrm{QPD}}_{4D}}\right)$$ (11)

where $\Delta L_{M/R}$ is the optical path length drift of the measurement and reference interferometers, *φ* represents the phase of the interferometric signals from photodetectors or the radio frequency (RF)-mixer signal from the acoustic-optic modulator (AOM) drivers, and Δ*L* is the final translation interferometric readout. *D_db_* is the distance between dual parallel sensing beams, and $\Delta \theta_{\mathrm{db}}$ is the corresponding tilt readout using one reflector in the experiment [32]. $\Delta \theta_{v/h}$ are the tilt readout of vertical and horizontal direction using the quadrant photodetector. *d* is the diameter of the beam incident on the sensitive area of single-element or quadrant photodetector. Equations 1/7 and 2/8 describe the optical path length drift of the measurement interferometer and the reference interferometer, respectively. Equation 3 represents the final translation interferometric readout of DBHI [33]. Equation 9 represents the final translation interferometric readout of PMHI. Equation 4 is the tilt readout expression using dual-beam interferometry [32]. Equations 5/10 and 6/11 describe the vertical and horizontal tilt readout using differential wavefront sensing [34].

### Test setup and environment

**Fig. 4.**
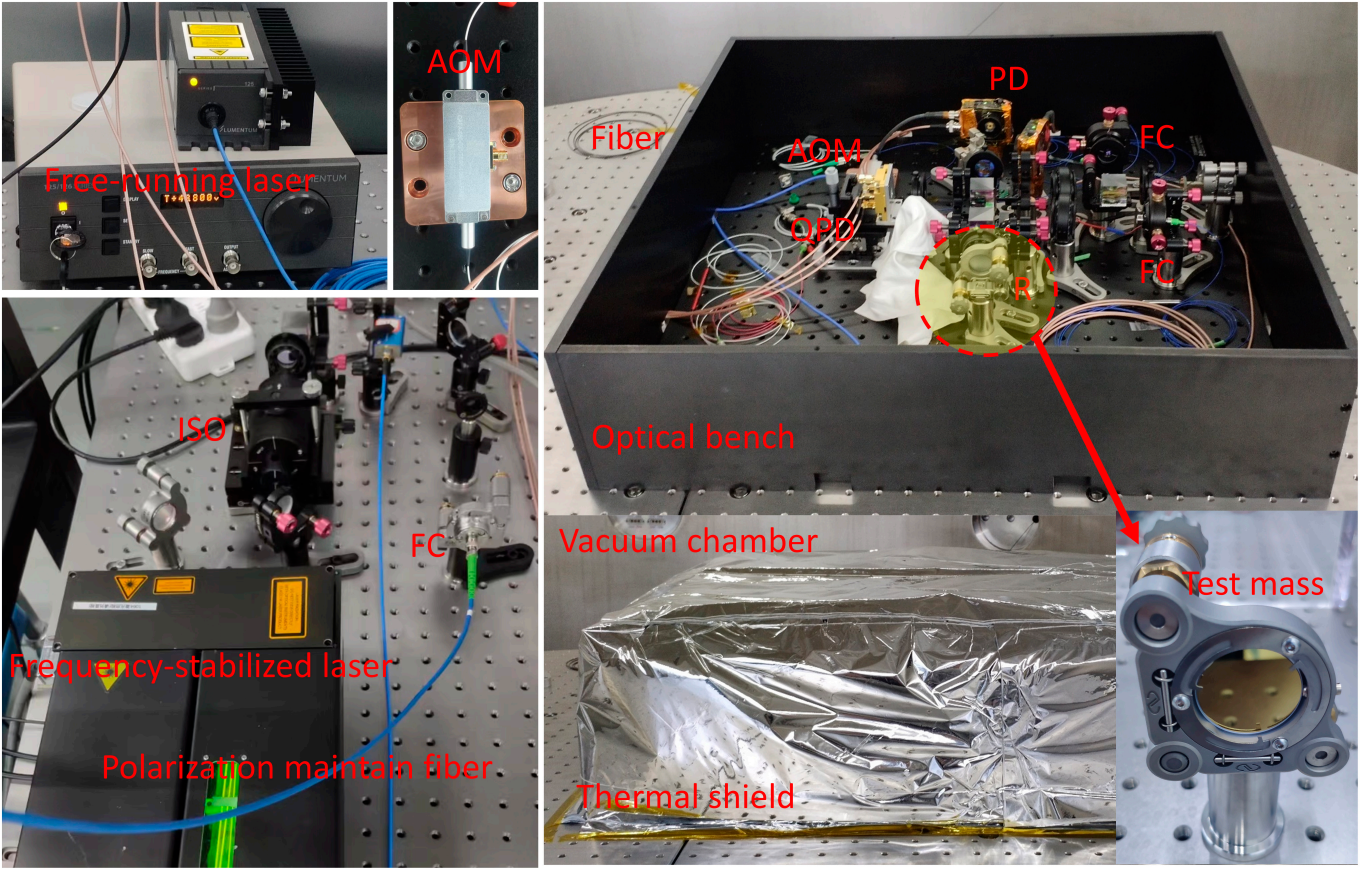
Laser heterodyne interferometric bench. AOM, acoustic-optic modulator; ISO, isolator; FC, fiber coupler; PD, photodetector; QPD, quadrant photodetector; R, reflector.

Initial experimental results of the interferometric readout yielded an optical path length drift of several nanometers, which can be attributed to the effects of the test environment perturbation, including air fluctuations and thermal and seismic noise in the setup. To enhance the measurement sensitivity, the experiments are then conducted in vacuum and various optimizations are employed. Figure 4 shows the physical setup of the system and the test environment. The center wavelength of the used lasers in our experiments is 1064 nm. The amplitude of the commercial laser can be modulated by a free-space AOM or front-panel BNC interface. Two heating sources, AOMs and photodetectors on the optical bench, are carefully wrapped in thermal insulation material and set in contact with the base to minimize temperature fluctuations. Most of the mechanical support components are made of stainless steel and tightly fixed to the aluminum breadboard, while the optical lenses are installed in detachable mountings that can be changed or recombined. Single-mode polarization-maintaining fibers and thin-film polarizers (colorPol VISIR CW02, Codixx) with an extinction ratio of 10^6^ are used in the bench, and the interferometric contrast is over 0.8 after careful alignment and adjustments. The unequal arm mismatch of the constructed DBHI and PMHI is ~0.5 mm and ~1 mm, respectively. During the testing, the vacuum degree of the testing environment is maintained at approximately 1 × 10^−2^ torr, and the entire optical bench is thermally shielded by an aluminum cover and multilayer heat-reflective film.

### Measurement sensitivity of the translation and the tilt

**Fig. 5.**
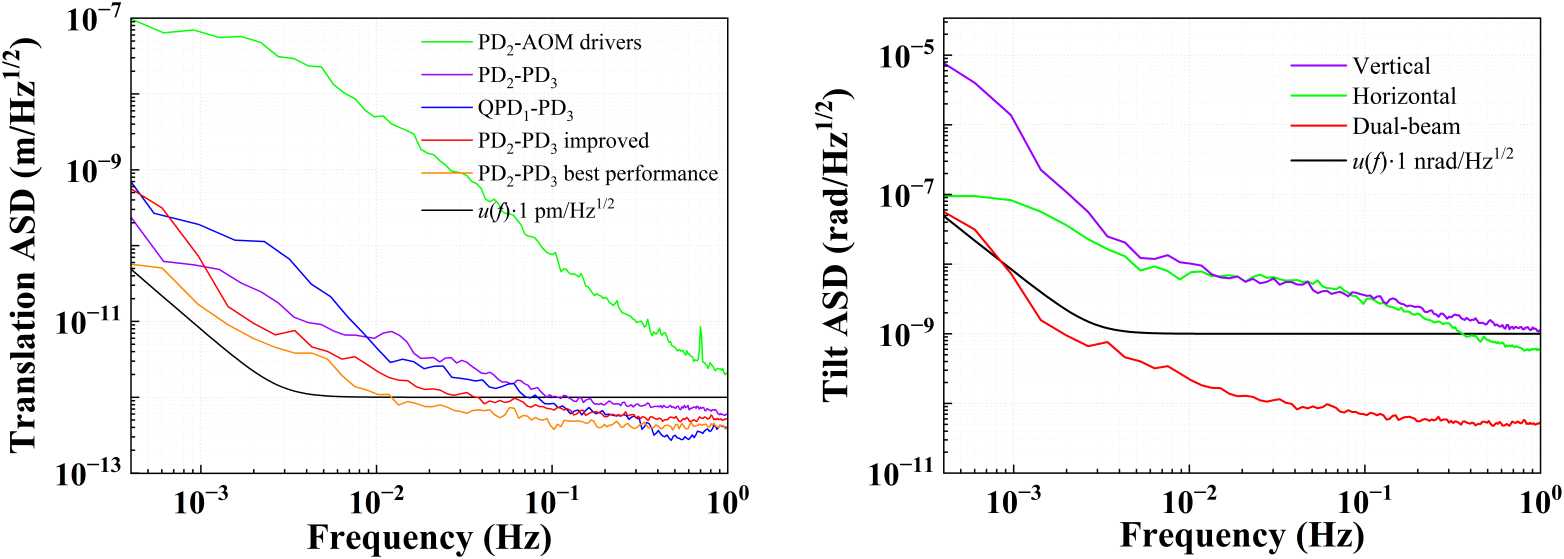
Measurement sensitivity results of the translation (left) and the tilt (right) by DBHI.

**Fig. 6.**
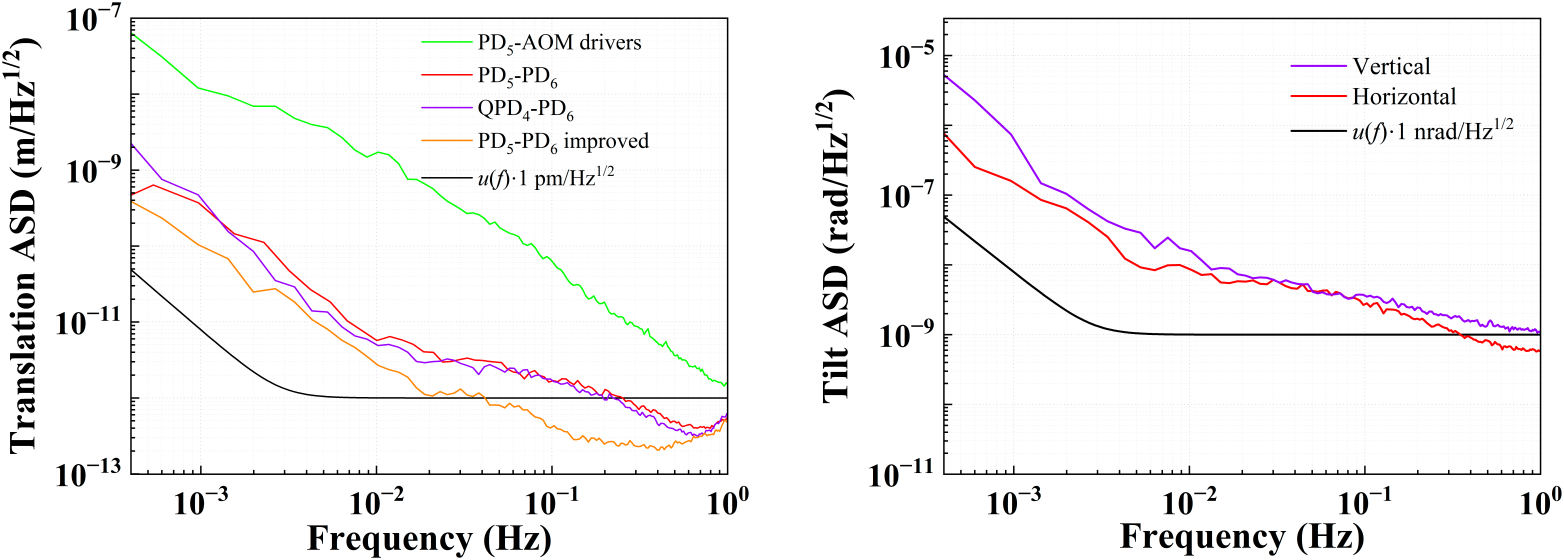
Measurement sensitivity results of the translation (left) and the tilt (right) by PMHI.

The crucial role of the laser interferometric bench in space-based gravitational wave detection is to measure the tiny translation and tilt. As such, the measurement sensitivity of the constructed bench, or the instrumental noise, defined as the readout amplitude spectrum density (ASD), has been tested with a mirror of high reflectivity at 1064 nm acting as the test mass, and the results of DBHI and PMHI are presented in Figs. 5 and 6. Note that all the spectral estimates in this paper, including this one, are generated using the lpsd algorithm [35].

For the translation measurement, which is the displacement motion along the measured beam, the sensitivity can reach 1 pm/Hz^1/2^ above 40 mHz by DBHI and PMHI using the single-element photodetector. However, the low-frequency translation sensitivity deteriorates, as the readout is 3 pm/Hz^1/2^ by DBHI or PMHI at 10 mHz. There is no improvement in the experimental results between using a frequency-stabilized laser and using a free-running laser. It is currently speculated that the long-term temperature fluctuations may have impacted the final readout. Overall, the test results could almost reach the baseline under the improved case of better linear polarization maintenance, and there is a difference of no more than one order of magnitude from the *u*(*f*)∙1 pm/Hz^1/2^ baseline [*u*(*f*) is the envelope function [17]] in the low-frequency band.

For the tilt measurement, which is also known as pitch or yaw, the constructed interferometric bench of DBHI and PMHI has a readout sensitivity of below 12 nrad/Hz^1/2^ for frequencies above 10 mHz using the quadrant photodetector. The tilt sensitivity of DBHI is greatly improved and well qualified below 1 nrad/Hz^1/2^ when utilizing the ratio of displacement between dual parallel sensing beams and their distance as the tilt readout. The distance is 10 mm in the experiments, determined by the lateral displacement beam splitter.

The further improvement of the measurement sensitivity is expected with higher vacuum degrees and better thermal insulation environment. Nevertheless, for many applications or technology verifications in the spaceborne gravitational detection missions [18,20,22,23], there is no strict limitation on the 1 pm/Hz^1/2^ and 1 nrad/Hz^1/2^ required measurement sensitivity of translation and tilt, typically viewed as a baseline for comparison or evaluation. The specific requirement of the measurement sensitivity depends on the targeted applications. Therefore, the obtained results demonstrate that the constructed bench with reconfigurable components has the potential for the practical use of related applications in the spaceborne gravitational wave detection missions, some of which we have verified in the following content.

## Discussion and Conclusion

### Implement of stabilization loops

In addition to the translation and tilt readouts, the technology to implement stabilization loops is also crucial in the development of spaceborne heterodyne interferometers [11,36–38]. Two stabilization loops are implemented in the constructed bench: the power stabilization of the amplitude and the optical path difference stabilization of the phase.

**Fig. 7.**
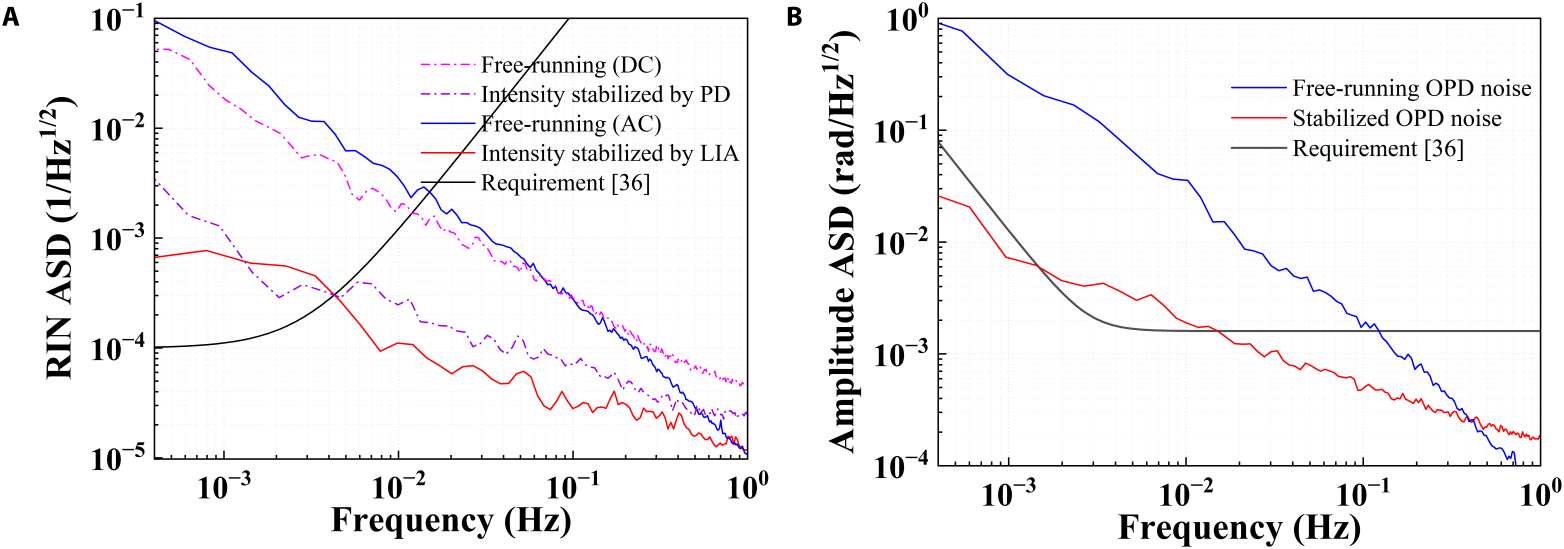
Implements of stabilization loops. (A) Power stabilization of the amplitude. (B) Optical path difference stabilization of the phase.

For the power stabilization of the amplitude, there are two methods to stabilize the laser amplitude drift. The first method involves using the AOM modulator, with the DC output of the photodiode power sensor as the error signal of proportion integration differentiation (PID) controller. Another method is using the direct interface of the free-running laser, with the AC amplitude output of the lock-in amplifier as the error signal. The relative intensity noise results are given in Fig. 7A, indicating that the laser amplitude noise can be greatly suppressed after applying the stabilization, which reaches 2.5 × 10^−4^ /Hz^1/2^ at 10 mHz.

For the optical path difference stabilization of the phase, the front-end phase drift has to be stabilized as this noise will couple into the final interferometric readout due to some nonlinear effects of stray light reflections and electronic noises from AOM drivers, which is known as small vector noise [38]. The workflow of optical path difference stabilization is the same as the laser amplitude, while the modulator here is a reflector driven by a piezoelectric actuator in the reference beam path and the error signal is from the phase of the reference interferometer. As depicted in Fig. 7B, the optical path difference noise is 2 × 10^−3^ rad/Hz^1/2^ at 10 mHz after stabilization, which is corresponding to 0.17 nm/Hz^1/2^.

### Coherence analysis

**Fig. 8.**
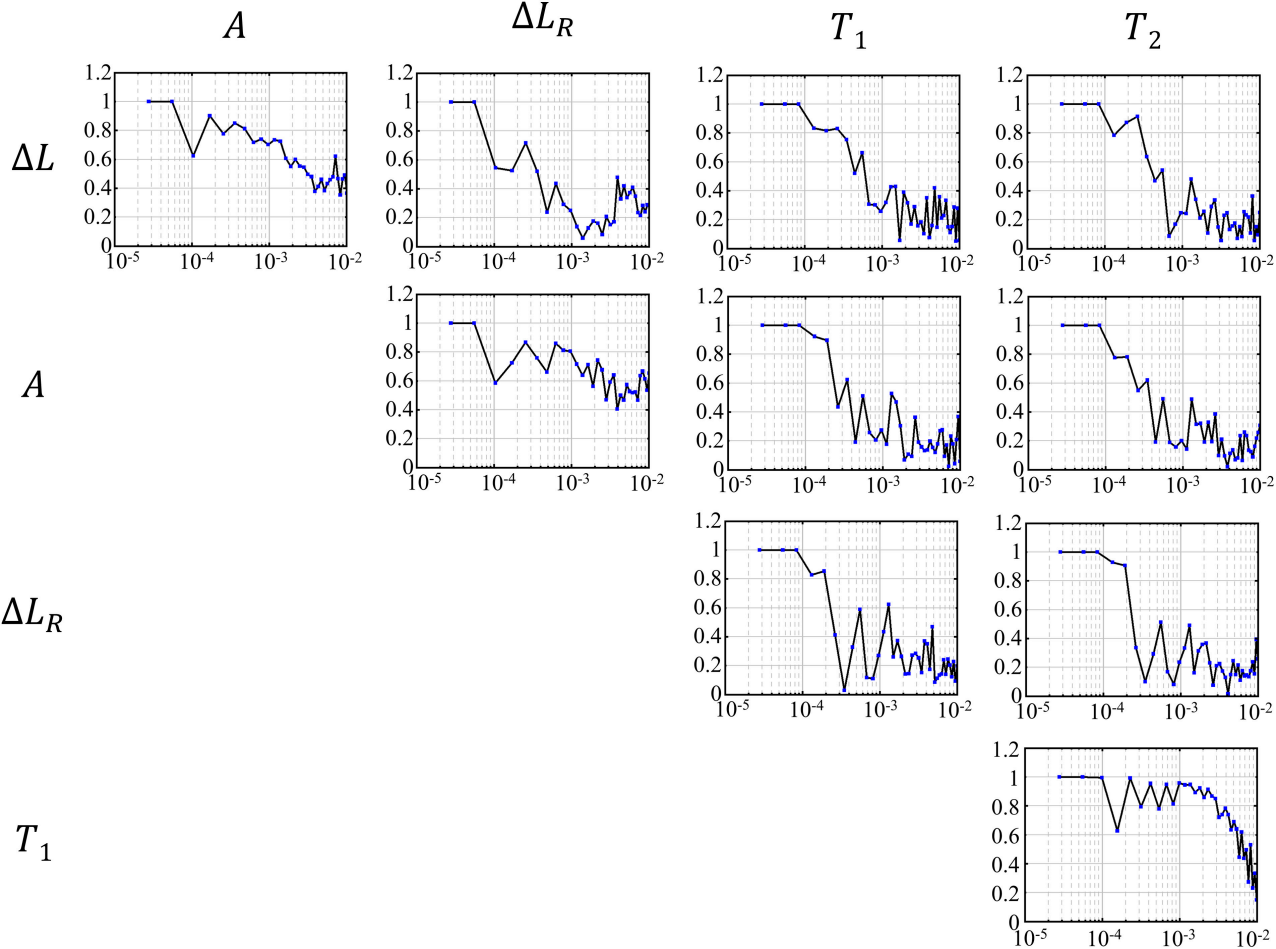
Coherence functions between different measurements. Units for the *x*’s values are in hertz. Δ*L* is the final translation readout. *A* is the amplitude of the heterodyne signal. $\Delta L_{R}$ is the optical path length drift of the reference interferometer. *T*_1_ and *T*_2_ are the temperature readout of two thermistors, which are placed in the thermal shield and the vacuum chamber, respectively.

Coherence between noise sources and the final interferometric readout has been reported in previous research as a means of mitigating noise and optimizing performance for the constructed interferometers [39]. Taking the temperature and the translation readout as an example, it is defined as follows:

$${\gamma^{2}}_{T_{i}\Delta L}\left(f\right)=\frac{{\left|S_{T_{i}\Delta L}\left(f\right)\right|}^{2}}{S_{T_{i}T_{i}}\left(f\right)S_{\Delta L\Delta L}\left(f\right)}$$ (12)

where $S_{T_{i}\Delta L}\left(f\right)$ is the cross-spectral density and $S_{T_{i}T_{i}}\left(f\right)$ and $S_{\Delta L\Delta L}\left(f\right)$ are the autospectral density. The coherence function quantifies the amount of correlation between two parameters of a linear system in each frequency bin, which allows us to evaluate the contributions from different noise sources to the final interferometric readout. In this paper, the correlations between the amplitude, the optical path length drift, the temperature, and the final translation readout with experimental results in 10 h are analyzed by the coherence function, and the results are shown in Fig. 8.

It should be noted that the results below 0.1 mHz are not effective since the Nyquist–Shannon sampling theorem. Nevertheless, for the translation readout, it reveals a strong correlation with the heterodyne signal amplitude in the low-frequency band (≃80% coherence at 0.4 mHz). Furthermore, the coherence analysis shows that the heterodyne signal amplitude and the optical path length drift are strongly correlated (≃70% coherence in the frequency band of 0.1 and 1 mHz). This suggests that the signal processing of amplitude and phase demodulation using the lock-in amplifier may be coupled with each other. The right two columns in Fig. 8 show the correlation between the temperature and other three parameters. It is believed that the temperature fluctuation has fair effects on the interferometric readout in the frequency band below 1 mHz. Moreover, it can also be told that the temperature in the thermal shield and in the vacuum chamber is strongly correlated (≃80% coherence) in the low-frequency band (*f* < 3 mHz). In future research, efforts will be made to enhance the stability of the laser amplitude and the front-end optical path length drift to see whether the performance of the final interferometric readout will get better. Another planned experiment involves designing a heating device to quantitatively evaluate the effects from the temperature fluctuations on the final readout of our laser heterodyne interferometric bench.

### Extended applications

**Fig. 9.**
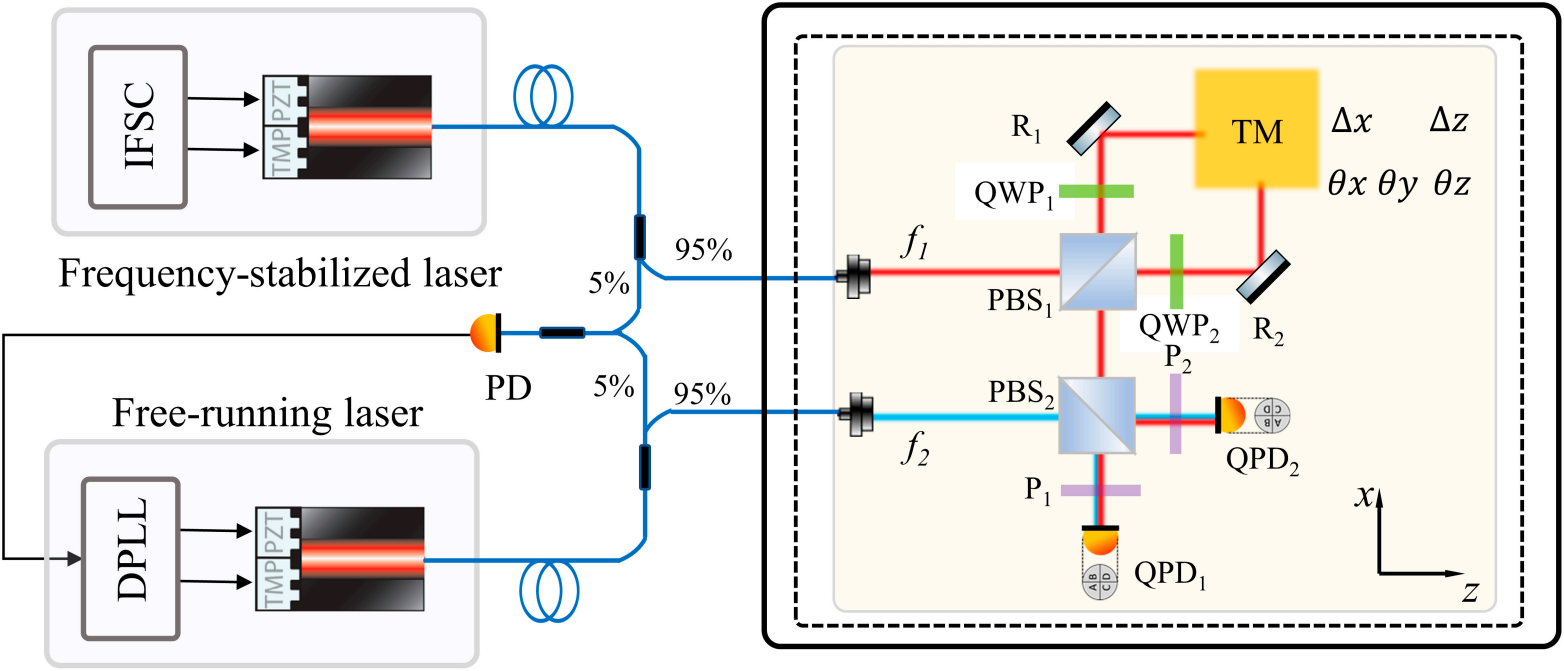
Design for extended applications of digital optical phase locking and multiple degree of freedom measurement. IFSC, iodine frequency stabilization controller; DPLL, digital phase-locking loop; TM, test mass.

A large number of optical benches have emerged to validate potential necessary technologies needed for the future spaceborne gravitational wave detection missions, such as deformation or surface diameter measurements [40], tilt-to-length noise analysis [18,19], and field-programmable gate array (FPGA) phasemeter testing [21]. Due to the employment of reconfigurable components, the constructed bench in the paper also has the ability to modify for extended applications. The design of two typical applications, the digital optical phase locking and multiple degree of freedom measurement, is shown in Fig. 9. The former is to generate two beams with a frequency difference by locking a free-running laser to a frequency-stabilized laser using the phase of the beat signal as the error input, while the latter can be used to monitor the motion of the freely falling test mass through the polarization-multiplexing design, which are both essential in the future spaceborne gravitational wave detection missions. The initial experimental details and results are presented in the Supplementary Materials, in which a heterodyne frequency locking and adjustment of 5 to 25 MHz through the constructed bench has been achieved. Based on the phase-locking technology, five degrees of freedom of the test mass has been measured combining the polarization multiplexing and differential wavefront sensing.

### Conclusion

In summary, the constructed laser heterodyne interferometric bench has demonstrated its sensitivity in both transitional and tilt measurements while also exhibiting noticeable flexibility of the setup, including laser amplitude and optical path length drift stabilization, coherence noise analysis, and extended applications of phase-locking and multiple degree of freedom measurements. The current measurement sensitivity of translation and tilt is 3 pm/Hz^1/2^ and 12 nrad/Hz^1/2^ for frequencies above 10 mHz, respectively, and would further promote testing in a more temperature-stable vacuum environment. The constructed laser heterodyne interferometric bench also has significant potential to remodify and verify key technologies for spaceborne gravitational wave detection missions of China, through which two extended applications of digital optical phase locking and multiple degree of freedom measurement have been demonstrated. Overall, construction and testing of the ultra-sensitive laser heterodyne interferometric bench demonstrates its potential to achieve a precise understanding of optical metrology in the spaceborne gravitational wave detection missions of China.

Moreover, high-precision heterodyne interferometry examined in this paper holds considerable significance in numerous industrial applications, such as extreme ultraviolet (EUV)-lithography, surface science metrology, and position control in nanofabrication. Therefore, the constructed bench can be used not only for the technology verification of spaceborne gravitational wave detection but also for several other high-precision industrial applications with reasonable modifications. In the future, performance optimizations of the laser interferometric bench, including noise analysis and its stabilization or elimination strategies, still need further study to push the boundaries of picometer-level translation and nanoradian-level tilt measurements. Other challenges to overcome in the spaceborne gravitational wave detection are also the focus of our future research, such as the laser frequency noise in the spaceborne long-arm interferometer and the tilt-to-length noise between two freely falling test masses, and we believe that the constructed bench would provide a competitive experimental platform with necessary performance.

## Materials and Methods

### Generating two beams with tiny frequency difference

The single-frequency laser beam in the heterodyne interferometric bench originates from the laser source unit, which consists of an iodine-stabilized nonplanar ring oscillator (NPRO) laser serving as the absolute reference. Two absolute reference lasers are available in our laboratory: one is from Prometheus by Innolight/Coherent and the other is from the National Institute of Metrology of China, in which the frequency stability can be tested by comparing two systems. Another fiber-coupling free-running laser (Lumentum, NPRO 125) is also available for the frequency and amplitude modulations. The beams are injected by a polarization-maintaining fiber into the optical bench and frequency-shifted by a pair of fiber-coupling AOMs manufactured by Castech Inc.

### Signal acquisition and processing

The heterodyne signals are acquired using two types of photodetectors, namely, two single-element photodetectors PDA10CS2 from Thorlabs Inc. and one customized quadrant photodetector. The signals are then transmitted to the management unit using cables of identical batch and length. As described in the “Results” section, the lock-in amplifier HF2LI from Zurich Instrument is employed for the amplitude and phase acquisition of the heterodyne signals, which are subsequently processed by LTPDA for data analysis.

### Temperature measurement system

The thermometer utilized in this paper comprises four PT1000 thermistors and one readout device (National Instruments, NI 9205). The readout sensitivity of the thermometer without the sensors can reach below 0.2 mK/Hz^1/2^ in the frequency band of 1 mHz and 1 Hz. The typical temperature measurement results under different conditions are presented in the Supplementary Materials.

## Data Availability

Data underlying the results presented in this paper are not publicly available at this time but may be obtained from the authors upon reasonable request.

## Supplementary Materials

Supplementary 1 Sections S1 to S3 Figs. S1 to S6
