# Supplementary material for: Verification of Laser Heterodyne Interferometric Bench for Chinese Spaceborne Gravitational Wave Detection Missions
Source: Research (Wash D C). 2024 Feb 14;7:0302. doi: 10.34133/research.0302 (PMC10865108; doi:10.34133/research.0302)
Supplement: Supplementary 1 — Sections S1 to S3 Figs. S1 to S6 [file research.0302.f1.zip › Supplementary Materials-R3.docx]

**Supplementary materials for**

Verification of laser heterodyne interferometric bench for Chinese spaceborne gravitational-wave detection missions

Xin Xu^1,2^, Heshan Liu^3^, Yidong Tan^1,2*^

*^1^ Department of Precision Instruments, Tsinghua University, Beijing 100084, China*

*^2^ State Key Laboratory of Precision Measurement Technology and Instruments, Tsinghua University, Beijing 100084, China*

*^3^ National Microgravity Laboratory, Institute of Mechanics, Chinese Academy of Sciences, Beijing 100190, China*

^*^Correspondence should be addressed to Yidong Tan: tanyd@tsinghua.edu.cn

**S1: Extended application of the constructed bench for laser phase locking.**

Traditional methods to generate the two beams with a slight frequency difference is to use a dual-frequency laser source of Zeeman effect, or combine a single-frequency laser source with a frequency-modulated device, such as acoustic-optic modulator (AOM) or electro-optic modulator (EOM). However, the dual-frequency laser source often has the problem of nonlinearity, which will cause several nanometer errors in one cycle of the half wavelength, which is obviously not suitable for the construction of optical bench for the spaceborne gravitational wave detection missions.

In many past studies, there often employs a pair of acoustic-optic modulator to generate space-separated dual-frequency beams during the test on the ground or in space. Nevertheless, it has been proved that the AOM drivers with poor quality will cause ‘shoulder’ noise in the translation measurement, and the power consumption and heat radiation will definitely make the optical system operating in the space more complicated.

Therefore, in such backdrop, the laser phase locking is proposed to handle the problem of dual-frequency laser sources in the space. The main principle is to lock one free-running laser to a frequency-stabilized laser with a slight frequency difference, which we often call the beat frequency. In the spaceborne gravitational wave detection missions, the range of the beat frequency is normally 5-25 MHz. Through the constructed bench, we have conducted the laser phase locking experiments, the optical design and the results are shown as below. A free-running laser is locked to the frequency-stabilized laser with a constant frequency difference, which can be adjusted within the range of 5-25 MHz as shown in the Fig. S2. The initial experiments demonstrate that the constructed bench can act as a platform to verify the laser phase locking technology, which is of significance for the future gravitational wave detection missions to provide a dual-frequency laser source.


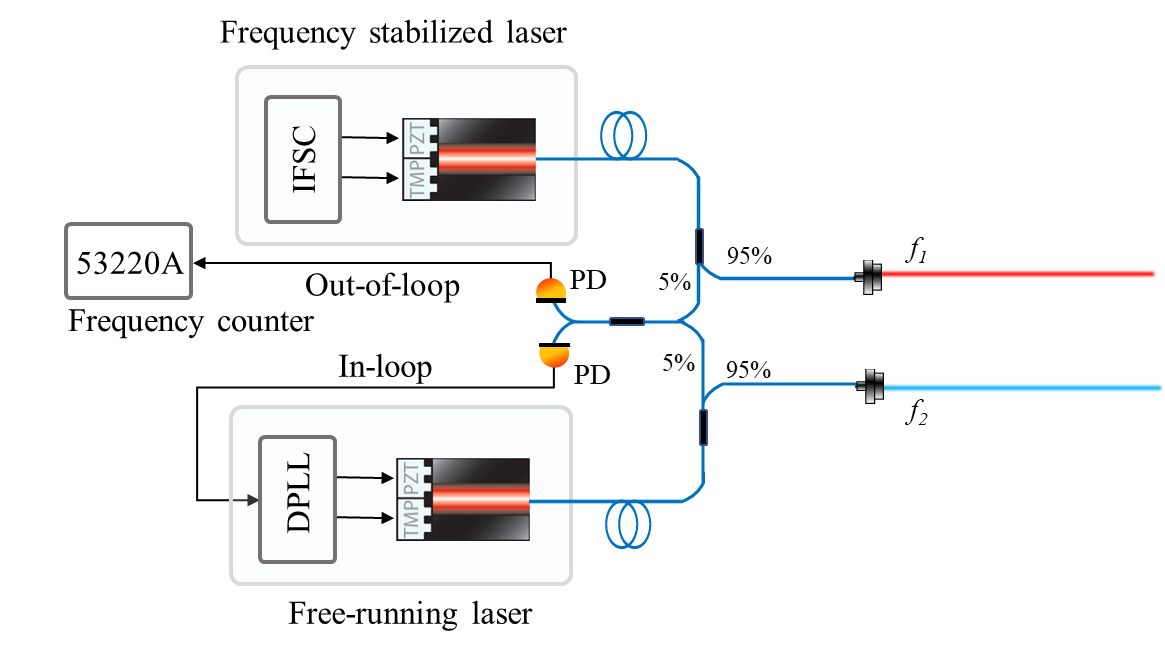


Fig. S1 Optical design of laser phase locking to generate dual-frequency beams.


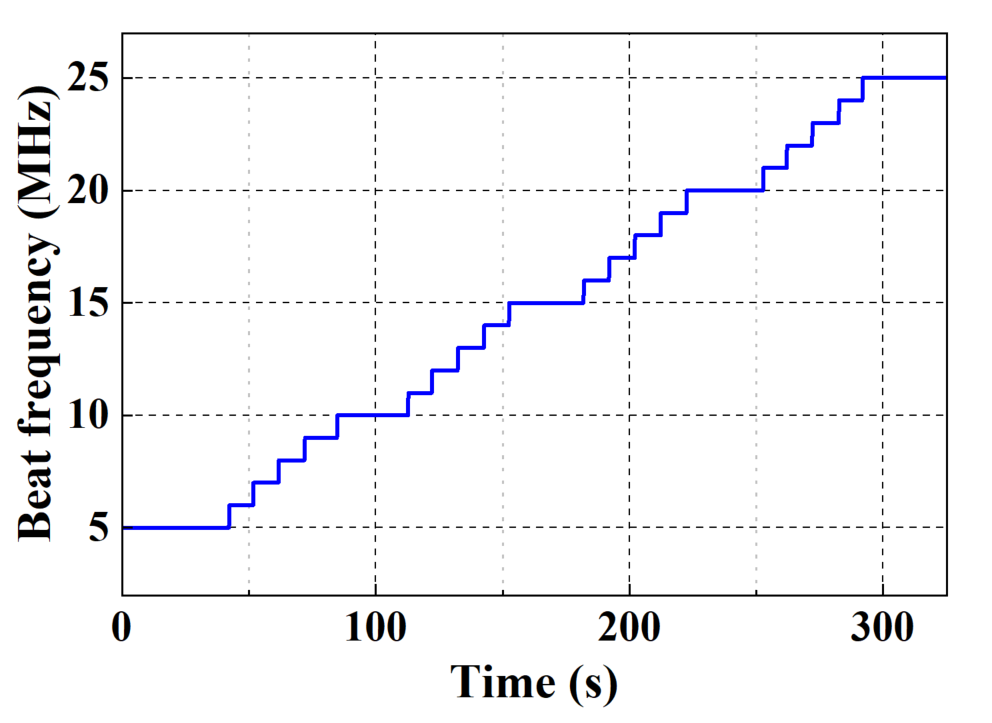


Fig. S2 The out-of-loop beat frequency results using the phase locking technology, recorded by a frequency counter (Keysight, 53220A).

**S2: Extended application of the constructed bench for multi-degree-of-degree measurements.**

The freely-falling test mass is the measured target in the final missions, the precise measurement of whose motions are vital for the successful beat-frequency signal acquisition. Thus, the precise measurement technology for the picometer and nanoradian level measurement of the translation and tilt for the test mass is needed during the development of spaceborne missions. Through the constructed bench, we have designed a optical path to measure five degrees of freedom for the test mass, the 2D and 3D diagrams of the optical path design are shown in Fig. S3.


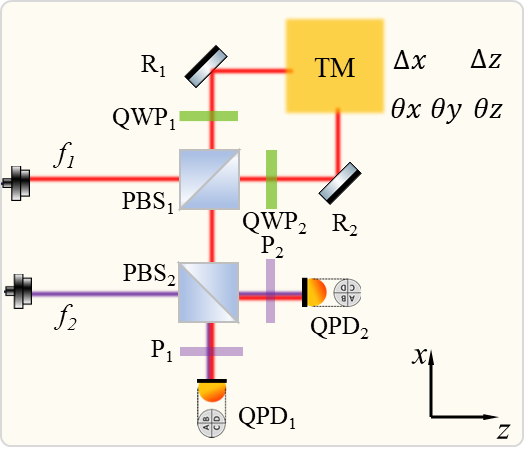

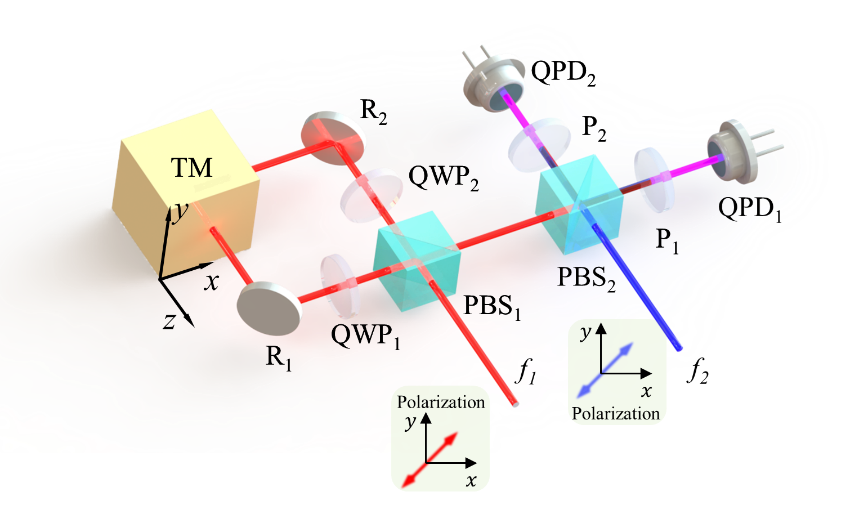


Fig. S3 The optical design of five degrees of freedom measurement through the constructed bench.

Fig. S1 illustrates the optical configuration of the polarizing-multiplexing heterodyne interferometric system. It contains two interferometers, each for the three degrees of freedom measurement using two quadrant photodetectors. Two beams (*f*_1_ and *f*_2_) with 45°linear polarization are incident and split by the polarizing beam splitter (PBS_1,2_). The detailed paths of the measurement beam and the reference beam for the two interferometers are listed in Table 1. Taking the second interferometer as an example, shown in Fig. 2, the reference beam of horizontal linear polarization is incident onto the quadrant photodetector (QPD_2_) after a polarizing beam splitter (PBS_2_) and a polarizer (P_2_). The measurement beam of the second interferometer passes through the polarizing beam splitter (PBS_1_), and the transmissive part is reflected by the reflector and the test mass. Twice passing through the quadrant wave-plate changes the polarizing direction of the backward beam, so this beam can be reflected by the polarizing beam splitter (PBS_1_). Then, the measurement beam is combined by the polarizing beam splitter (PBS_2_) with the reference beam. The polarizer (P_2_) keeps the same polarizing part and the beat signals are detected by the quadrant photodetector (QPD_2_) with the active area divided into the quadrants A-B-C-D.

To verify the feasibility, we use a nanometer-precision displacement stage (Physik Instruments, P-562.6CD) to load a reflector with five-degree-of-freedom motions. The resolution and range test results are shown in the Fig. S4, showing that the constructed bench has the ability changeable for the multi-degree-of-freedom measurements.


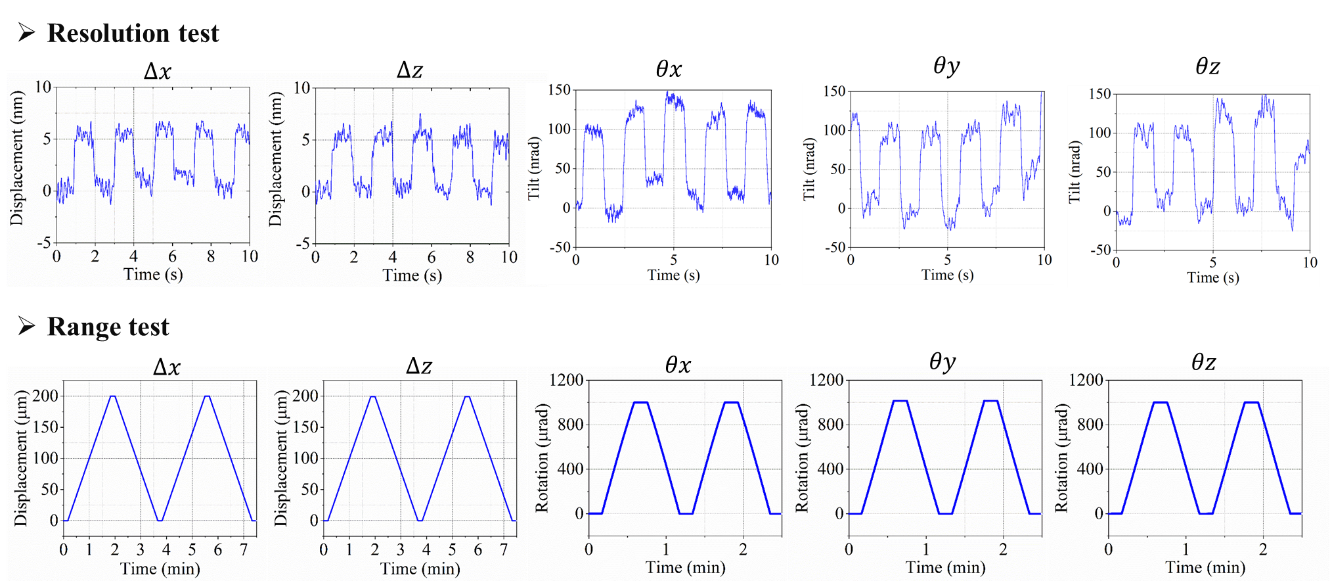


Fig. S4 Five degrees of freedom through the constructed bench.

**S3: The temperature measurement device and the test results under different conditions.**

The initially developed thermometer is made of four thermal resistances (PT1000), a data acquisition module and the labview display unit, which are shown in the Fig. S5. In the current condition of vacuum environment, the temperature stability of the constructed bench can reach 0.6 mK/Hz1/2 at the frequency of 1 mHz. The test results of the high-frequency band above are dominated by the electronic noises from the developed thermometer itself, which cannot measure the actual temperature of the tested vacuum environment. Nevertheless, the developed thermometer can provide the temperature results for the coherence analysis to show the relevance in the frequency below 2 mHz.


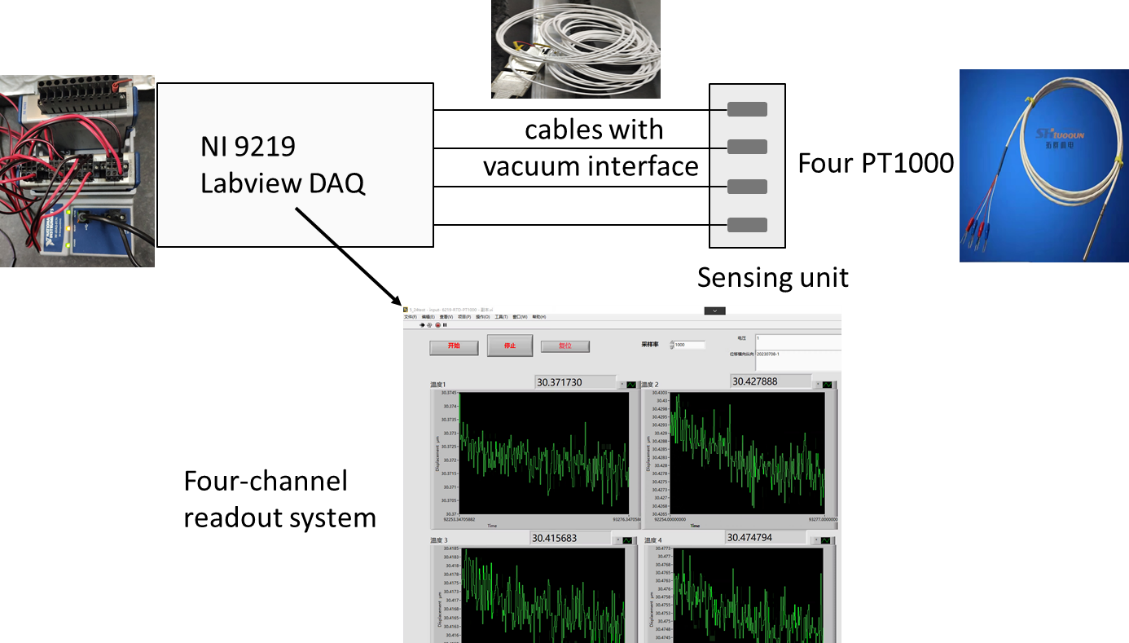


Fig. S5 Four-channel temperature measurement system


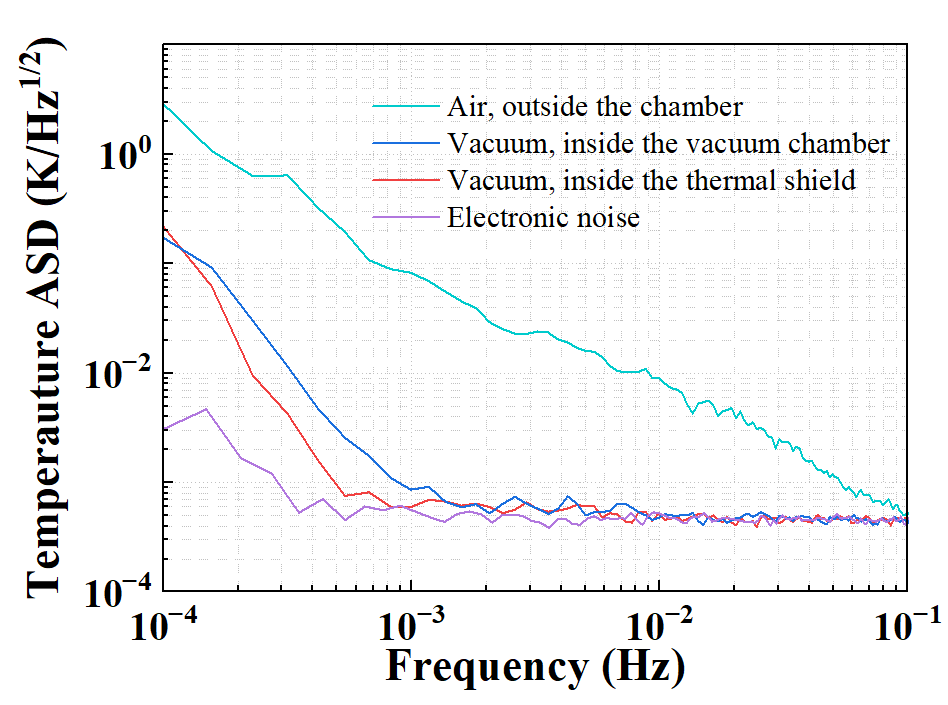


Figure. S6 The temperature measurement results. The electronic noise curve represents the limitation readout using the developed thermometer.
