# Supplementary figures and images for: Verification of Laser Heterodyne Interferometric Bench for Chinese Spaceborne Gravitational Wave Detection Missions
Source: Research (Wash D C). 2024 Feb 14;7:0302. doi: 10.34133/research.0302 (PMC10865108; doi:10.34133/research.0302)

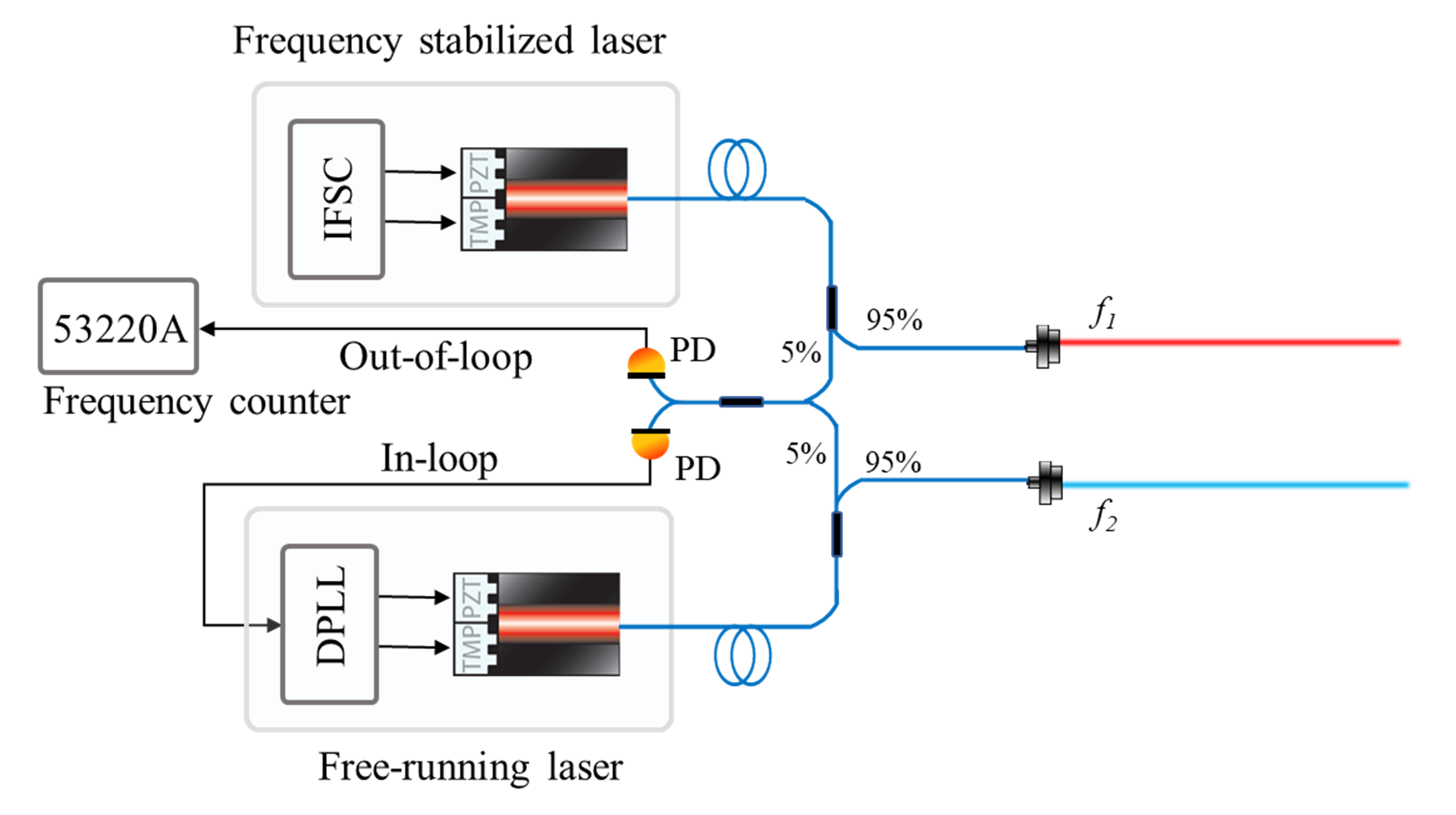

Supplement: Supplementary 1 — Sections S1 to S3 Figs. S1 to S6 [file research.0302.f1.zip › figure S1.tif]

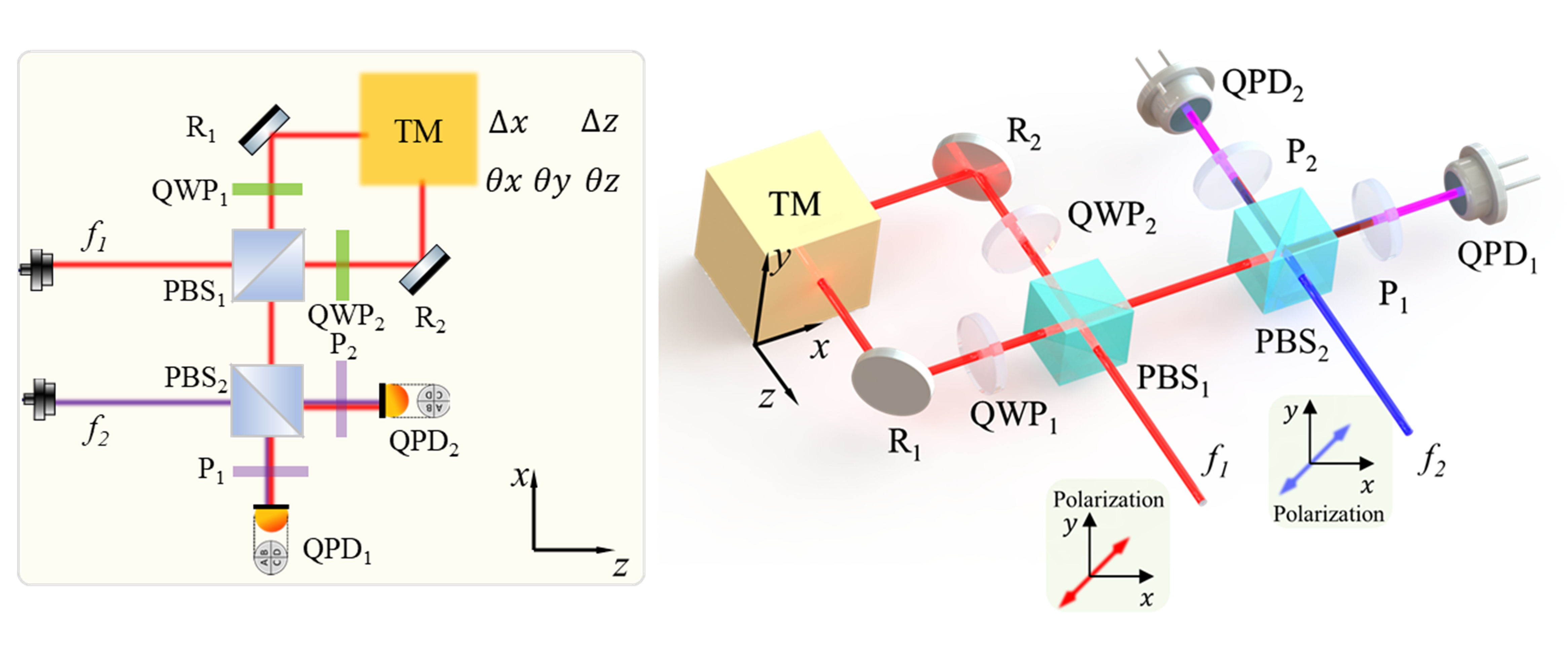

Supplement: Supplementary 1 — Sections S1 to S3 Figs. S1 to S6 [file research.0302.f1.zip › figure S3.tif]

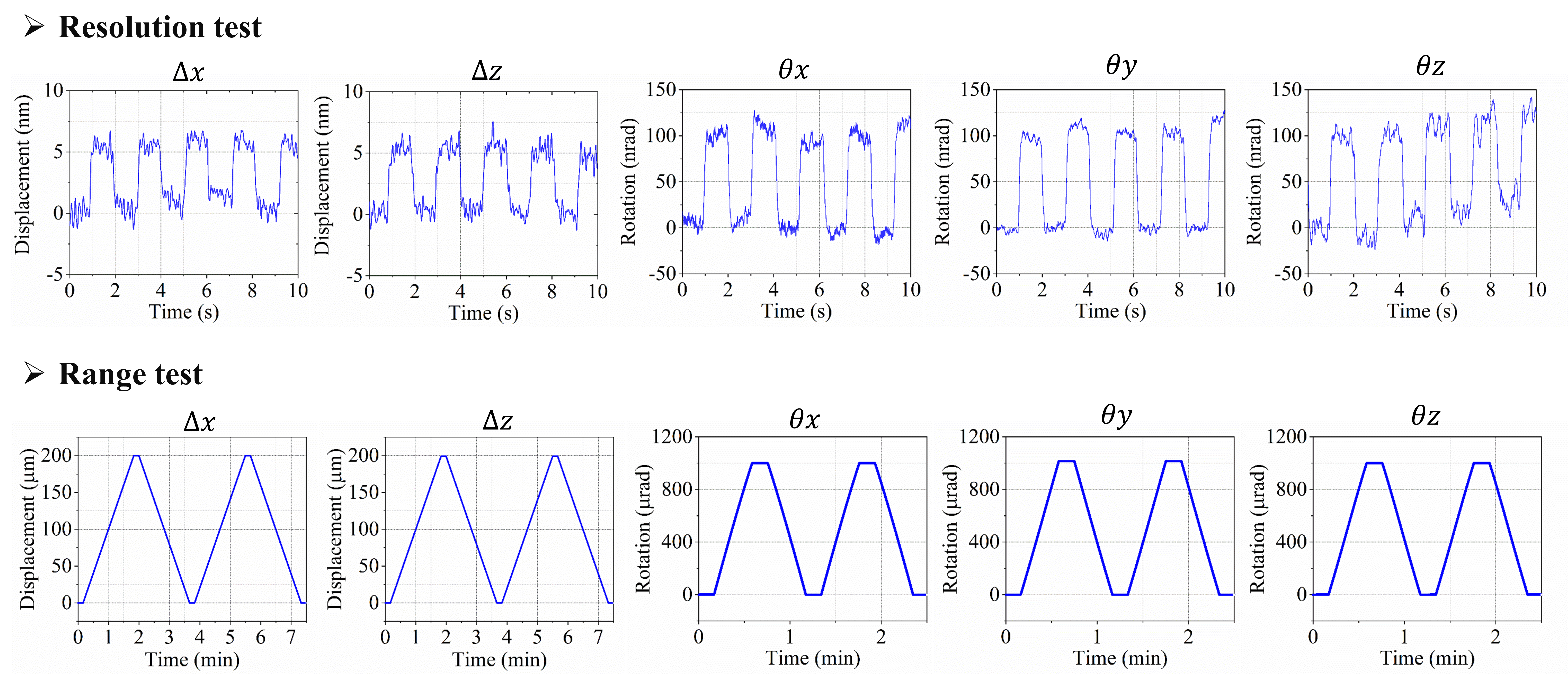

Supplement: Supplementary 1 — Sections S1 to S3 Figs. S1 to S6 [file research.0302.f1.zip › figure S4.tif]

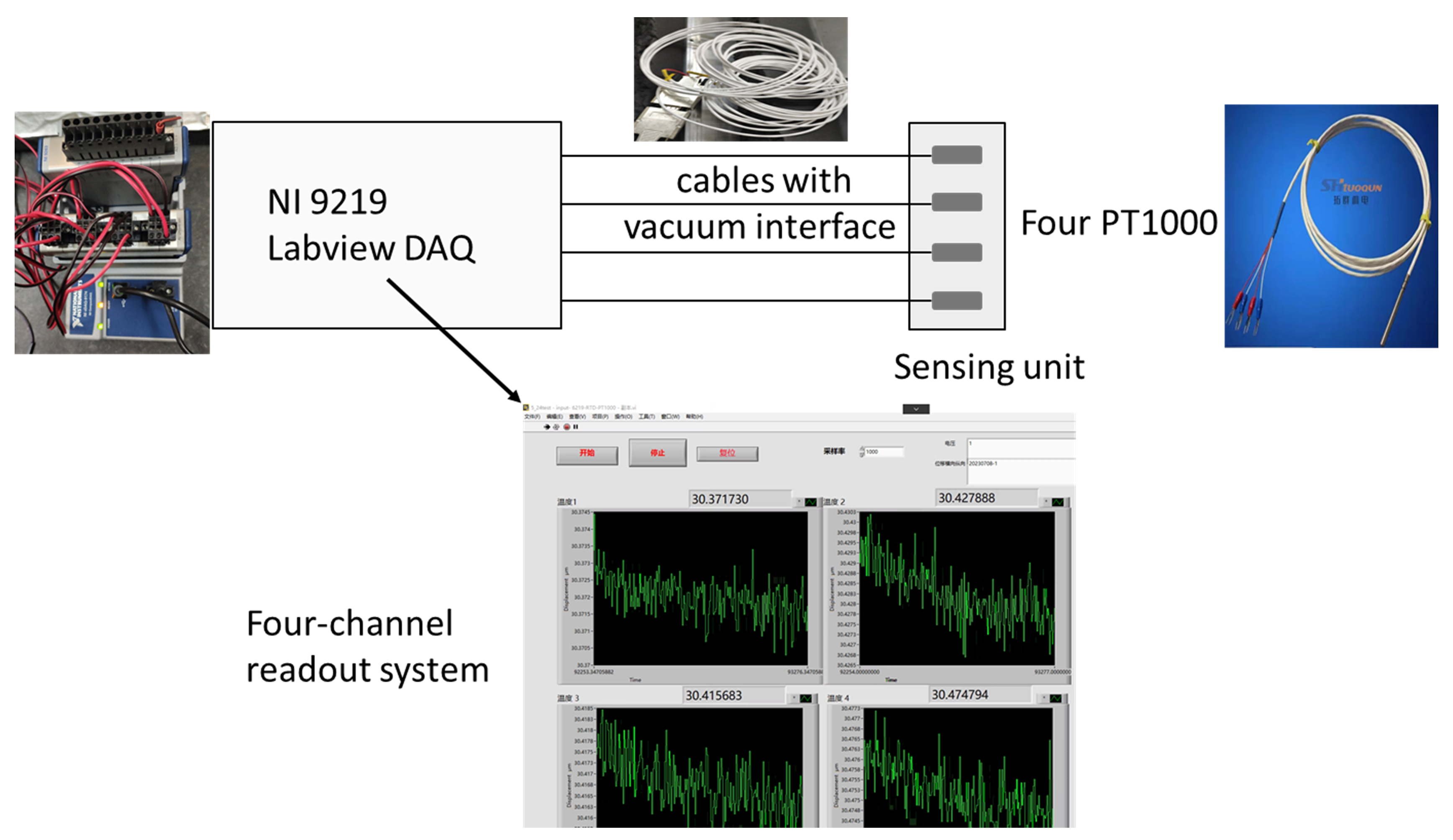

Supplement: Supplementary 1 — Sections S1 to S3 Figs. S1 to S6 [file research.0302.f1.zip › figure S5.tif]
